# Classics in Chemical Neuroscience: Tianeptine

**DOI:** 10.1021/acschemneuro.4c00519

**Published:** 2024-10-09

**Authors:** Yu Nishio, Craig W. Lindsley, Aaron M. Bender

**Affiliations:** ^†^Warren Center for Neuroscience Drug Discovery, ^‡^Department of Pharmacology, ^§^Department of Chemistry, and ^∥^Department of Biochemistry, Vanderbilt University, Nashville, Tennessee 37232, United States

**Keywords:** tianeptine, antidepressant, opioid, serotonin, drug abuse

## Abstract

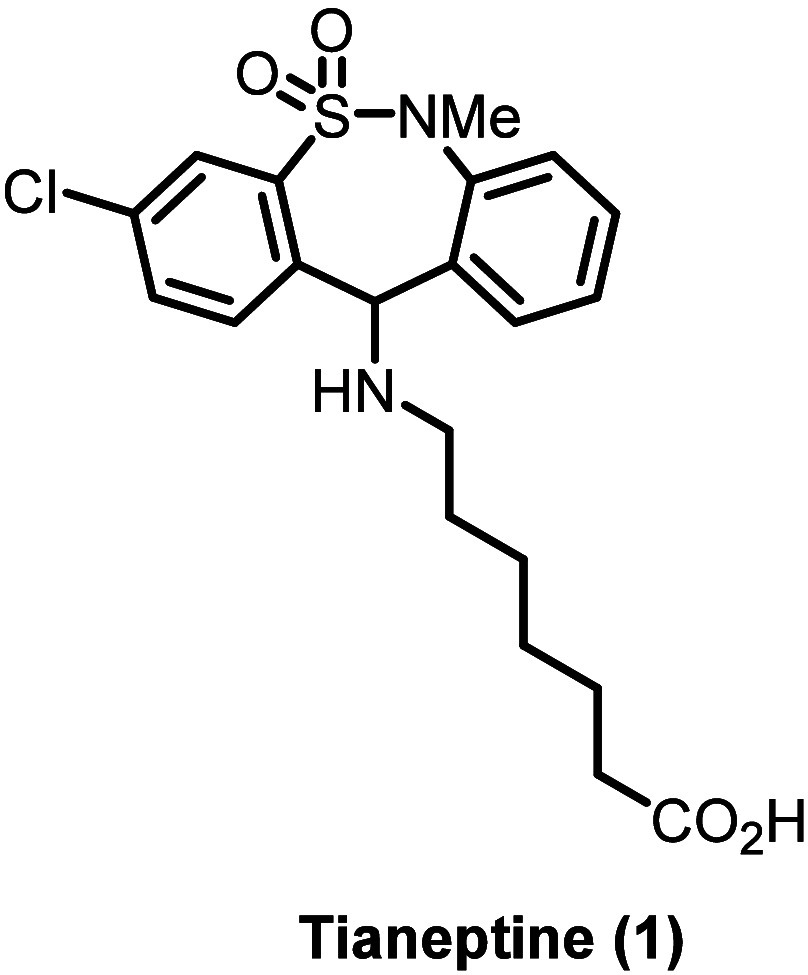

Tianeptine (**1**) is an unusual antidepressant in that its mechanism of action appears to be independent from any activity at serotonin receptors or monoamine transporters. In fact, tianeptine has been shown to be a moderately potent agonist for the mu opioid receptor (MOR) and to a lesser extent the delta opioid receptor (DOR). Additionally, tianeptine’s efficacy may be related to its action on glutamate-mediated pathways of neuroplasticity. Regardless of which neurotransmitter system is primarily responsible for the observed efficacy, the MOR agonist activity is problematic with respect to abuse liability. Increasing numbers of case reports have demonstrated that tianeptine is indeed being used recreationally at doses far beyond what are considered therapeutically relevant or safe, and scheduling reclassifications or outright bans on tianeptine products are ongoing around the world. It is the aim of this review to discuss the medicinal chemistry and pharmacology of tianeptine and to summarize this intriguing discrepancy between tianeptine’s historical use as a safe and effective antidepressant and its emerging potential for abuse.

## Introduction

Depression is one of the most common mental disorders globally. According to the World Health Organization (WHO), approximately 3.8% of the population experiences depression during their lifetimes.^1^ Due to the long-lasting symptoms of depression, which include decreased concentration, fatigue, anxiety, and suppression of mood, depression is a major contributor to suicide (which is the fourth leading cause of death in young adults). In addition, depression is a common comorbidity in a broad group of other disorders including Parkinson’s disease (PD) and asthma.^2,3^

**Figure 1:**
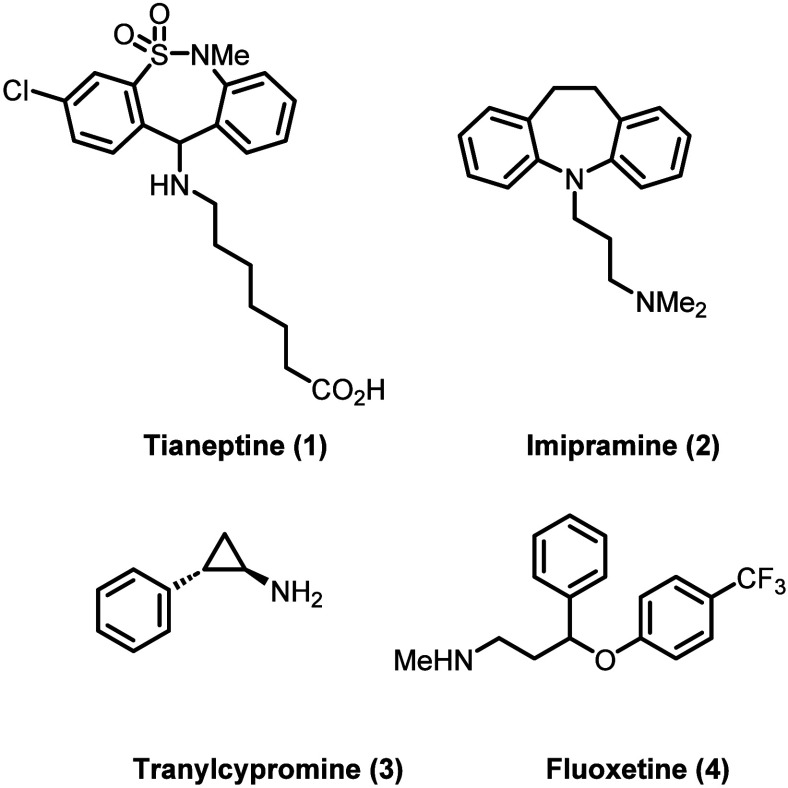
Chemical structures of tianeptine and selected FDA-approved antidepressants.

The global prominence of depression and the lack of a cure have spurred a vast number of drug discovery efforts. Monoamine oxidase inhibitors (MAOIs), tricyclic antidepressants (TCAs), and selective serotonin reuptake inhibitors (SSRIs) have all been extensively studied from the mid to late 1900s. The serotonin (5-hydroxytryptamine, 5-HT) hypothesis of depression put forth by Alec Coppen and others became an early guiding principle for medicinal chemists working in this space,^4−6^ and the echoes of this principle are apparent in the structural design of many marketed antidepressants including imipramine (**2**, TCA), tranylcypromine (**3**, MAOI), and fluoxetine (**4**, SSRI; see Figure 1 for chemical structures).^7−9^

Tianeptine (**1**, Figure 1), first synthesized in 1971 by the Science Union Et Cie, Societe Francaise de Recherche Medicale as an analog of the recently discovered TCAs, is something of a mechanistic outlier compared to the other major classes of antidepressants.^10^ Unlike SSRIs and structurally related TCAs, tianeptine was initially shown to *enhance* uptake of 5-HT in vivo without apparent activity at any serotonin receptors or monoamine transporters, although its mechanism of action may well be unrelated to serotonergic neurotransmission (see the pharmacology sections for a more detailed discussion).^11^

Tianeptine (brand names include Coaxil, Stablon, and Tatinol) is now used as an antidepressant in over 60 countries around the world (predominantly in Europe, Asia, and South America) and is not currently approved for use in the United States (among other countries including Canada and Australia).^12,13^ Despite this lack of FDA approval, tianeptine is easily obtained in gas stations and convenience stores and through online retailers. The compound is commonly sold through these channels under names including, but not limited to, Zaza, Tia, Tianna, Zaza Red, TD Red, and Pegasus; tianeptine is also colloquially known as “gas station heroin”.^14^ Recreational misuse of tianeptine can lead to opioid-like symptoms, such as an increase in dopamine levels, addiction, and respiratory depression.^15,16^ Cases of accidental overdose and intentional use in suicide have also been documented,^17^ and an overall increase in cases of tianeptine use has been reported worldwide, particularly in the United States and Europe. In the United States, the National Poison Data System (NPDS) reported 11 tianeptine exposure calls in the years between 2000 and 2017 and 207 calls between 2014 and 2017, and the FDA reported 151 cases in 2020.^18−20^ Due to this increase, some states have banned products containing tianeptine and have designated it a Schedule I or II controlled substance.^21^

## Chemical Properties and Synthesis

### Chemical Properties

**Scheme 1:**
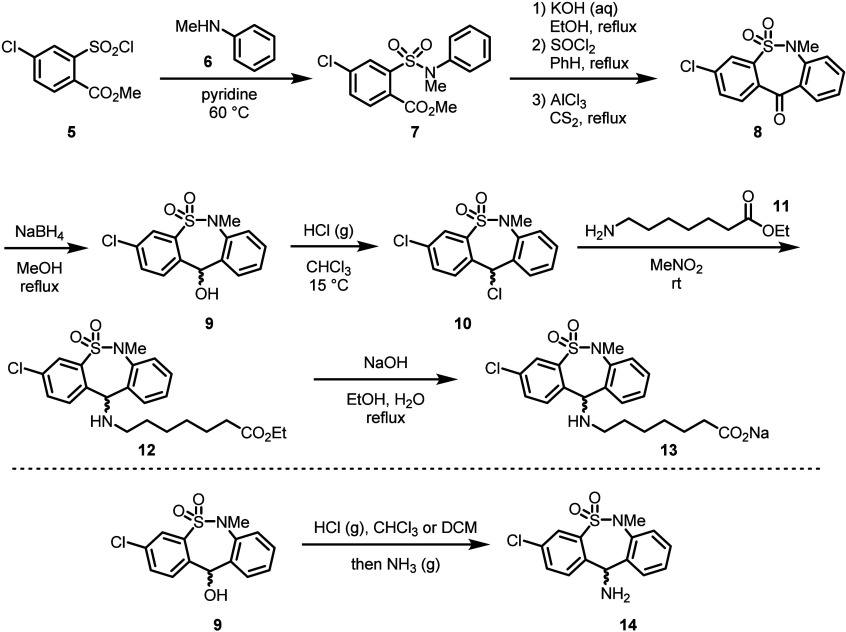
Initial Synthesis of Tianeptine (Top), and Modification of the Synthesis for Improved Yield (Bottom)

Tianeptine (CAS no. 72797-41-2; IUPAC: 7-[(3-chloro-6-methyl-5,5-dioxido-6,11-dihydrodibenzo[*c*,*f*][1,2]thiazepine-11-yl)amino]heptanoic acid, C_21_H_25_ClN_2_O_4_S) has an interesting tricyclic structure with a resemblance to that of other TCAs. It differs from the conventional TCAs, however, by the seven-membered sultam ring system and carboxylic acid tail (Scheme 1). Tianeptine has two hydrogen bond donors and six hydrogen bond acceptors and has a molecular weight of 436.953 g/mol. Tianeptine is amphoteric (p*K*_a_ = 4.4 (acidic) and p*K*_a_ = 6.86 (basic)) with a logP of 1.06 at pH 7.4, making the molecule compatible with Lipinski’s rule of five.^22,23^ Tianeptine (as the free carboxylic acid) has a melting point of 144–147 °C and is a white powder, while the sodium salt has a melting point of 148 °C and is generally encountered as a white to pale yellow powder.^24,25^ Tianeptine as well as the sodium salt (CAS no. 30123-17-2) are commercially available from a variety of chemical vendors.

**Figure 2:**
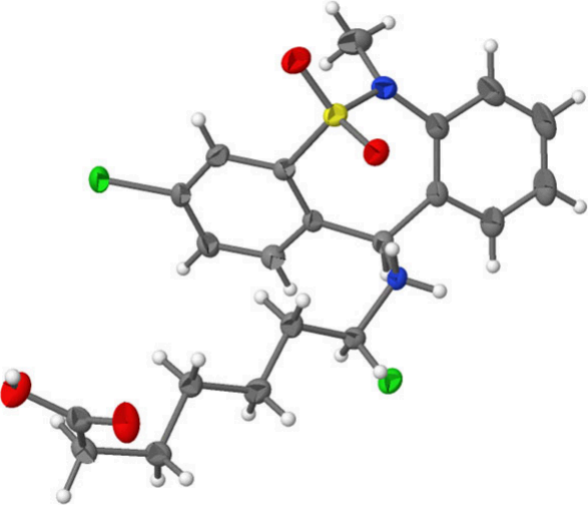
X-ray crystal structure of tianeptine hydrochloride. Reprinted with permission from ref (26). Copyright 2012 IUCr Journals.

An X-ray structure of the hydrochloride salt revealed the seven-membered sultam ring adopts a boat-type conformation, and a dihedral angle between the mean planes of the benzene rings of 44.44(7)° was observed. Interestingly, an intramolecular hydrogen bond between a sultam oxygen and the amino nitrogen was also observed (Figure 2).^26^

### Synthesis

The initial synthesis of the tianeptine core was reported in 1970 by the Science Union Et Cie, Societe Francaise de Recherche Medicale. A separate synthesis of the tail component was disclosed in an additional patent in 1971.^10,27,28^ In the first step of the core synthesis, nucleophilic substitution with aniline **6** onto the aryl sulfonyl chloride **5** formed the corresponding aryl sulfonamide.^29,30^ Then, hydrolysis of the ester, chlorination to give the intermediate acyl chloride, and finally Friedel–Crafts acylation resulted in tricyclic intermediate **8**. Reduction of the ketone and chlorination gave the tianeptine core **10** as the secondary alkyl chloride. Finally, nucleophilic substitution using ethyl 7-aminoheptanoate (**11**) and ester hydrolysis afforded tianeptine as the sodium salt (**13**).^10^

Various incremental improvements have been made to the synthesis of tianeptine to increase the yield and purity; next-generation efforts have primarily focused on the ethyl amino heptanoic acid tailpiece due to the instability of ethyl 7-aminoheptanoate in the reaction medium. Direct addition of the amine moiety to the tianeptine core to give intermediate **14** provided much higher yield and purity for large-scale synthesis.^31,32^ In addition, various functional group interconversions (FGIs) have been established to install the heptanoic acid tail in an efficient manner.^33^

### Medicinal Chemistry and SAR

**Figure 3:**
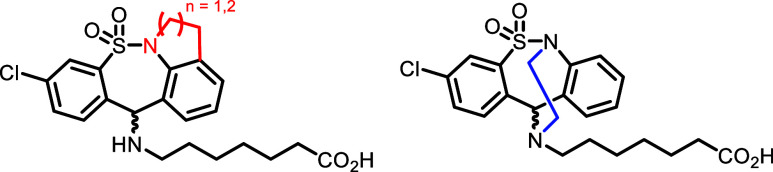
Selected recent examples of known modifications to the tianeptine scaffold.

In recent literature, analogues of tianeptine have been synthesized from the Directorate of Drug Substance Development, Egis Pharmaceuticals Plc. with focus on modifications to the *N*-methyl moiety.^34,35^ The aim was to modify the *N*-methyl moiety to block *N*-demethylation, a known metabolic pathway for tianeptine (see the Drug Metabolism and Pharmacokinetics section for further details). Thus, tetracyclic analogues linking the sultam nitrogen to the aromatic ring (red) and amine tail (blue) were synthesized in turn (Figure 3).

Due to the structural similarities between tianeptine and the TCAs, many of the SAR investigations for tianeptine have been reported as comparison studies with the latter, despite likely differences in therapeutic mechanisms. Because of the large numbers of reported TCA core scaffolds, there has historically been some difficulty in defining a precise set of SAR generalizations with respect to target potency. While unique SARs for specific TCAs have been studied, grouping multiple TCAs into one SAR model can be misleading due to the variety of biochemical mechanisms/pathways of these clinical TCAs.

Regardless, an initial study of tianeptine SAR was conducted with an eye toward historical TCAs, specifically an examination of the reversal of reserpine-induced ptosis potency in mice.^36^ The study focused primarily on four structural aspects of tianeptine: (1) the carbon count of the heptanoic acid side chain, (2) the terminal carboxylic acid functional group, (3) the chlorine substituent on the aromatic ring, and (4) the nature of the tricyclic system. The results of each of these separate scaffold modifications are summarized as follows.

**Figure 4:**
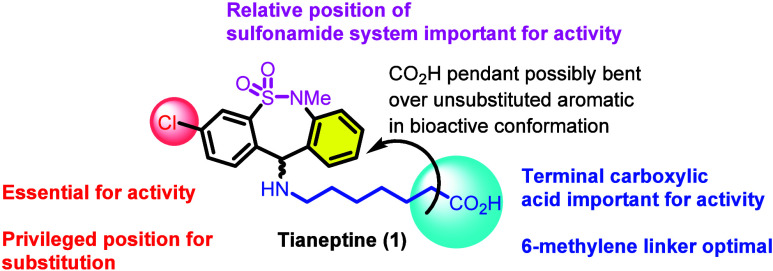
Tianeptine SAR from reserpine-induced ptosis.

(1) The optimal length of the heptanoic acid side chain was found to be 6 methylene linkers (as in the structure of tianeptine itself; deviation of this count by either adding or removing carbon spacers was found to be detrimental). Additionally, the installation of methyl groups to the side chain did not appreciably change activity. (2) The carboxylic acid functional group was shown to be crucial for tianeptine’s activity in this context. Alkyl chains and imipramine-like tertiary amine tails were not tolerated. (3) The position of aromatic ring substitutions was also found to be important for activity in this context; aromatic substitution at C_3_ was found to be essential for maintenance of activity. Various substitutions for chlorine were also examined at the C_3_ position, but chlorine itself was ultimately found to have the optimal activity. Additionally, because of the loss of activity observed by adding substituents to C_9_ (eastern aromatic ring, as drawn), it was hypothesized that the side chain is bent toward the unsubstituted aromatic ring in the relevant bioactive conformation, a proposal that has been corroborated by variable-temperature NMR studies.^37^ (4) An electron-donating heteroatom at C_5_ (the sulfonamide S atom) is required for activity (see Figure 4 for summarized findings).

After the finding that tianeptine has agonist activity at both the mu and the delta opioid receptors (MOR and DOR, see the pharmacology sections for further details), an additional study on the SAR of tianeptine was performed by screening for activity at the three human opioid receptor subtypes (MOR, DOR, and the kappa opioid receptor (KOR)) using a G protein BRET assay.^38^ Based on recent X-ray crystal structures and 3D modeling, the sulfonamide functional group was theorized to be important in this context as well.^26,39^ As in the previous SAR study, tianeptine analogs were generated with a primary focus on structural modifications to the side chain and substitutions on the aromatic core.^38^

**Figure 5:**
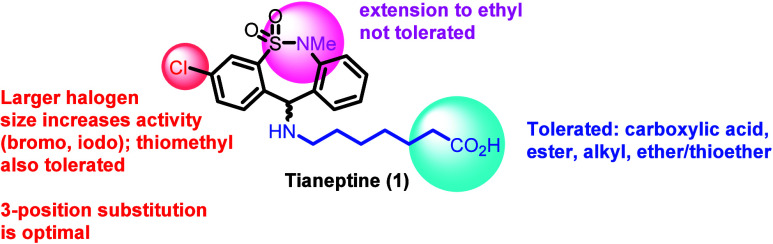
Summary of SAR findings for tianeptine and analogs at the MOR.

In contrast to the antireserpine-based SAR, a variety of modifications to the side chain were tolerated with respect to MOR activity (tianeptine itself is only very weakly DOR active, and the majority of analogs are either similarly weak or inactive). It was found that ester analogs of both tianeptine and one of its primary metabolites, MC_5_ (see the Drug Metabolism and Pharmacokinetics section for additional structural details), showed only a slight reduction in MOR potency compared to the parent analogs. Additionally, although both tianeptine and metabolite MC_5_ were found to be active at MOR, MC_5_ was found to be inactive at DOR. If MC_5_ plays any role in the antidepressant effects of tianeptine, it is therefore unlikely that these contributions are DOR-mediated. A variety of alkyl and ether/thioether functional groups displayed similar or only slightly diminished MOR potencies compared to tianeptine; however, the MOR potencies of the alcohol and aromatic derivatives were robustly diminished. Additionally, and in contrast to the reserpine study, larger halogens at the 3 position (bromo, iodo) were found to improve activity relative to the chloro substitution in the context of multiple side chains. Substitutions at other positions on the aromatic ring decreased activity; as in the previous reserpine-induced ptosis SAR study, the 3 position was shown to be a privileged position for functionalization on the aromatic rings. Extensions of the sulfonamide *N*-substitution (*N*-ethyl) were counterproductive (see Figure 5 for summarized findings).^38^

Tianeptine is typically encountered and prescribed as a racemic mixture of enantiomers at the side chain linkage position, and at present, it is unclear how each enantiomer differentiates with respect to opioid receptor binding (preliminary docking experiments suggest only marginal differences in MOR binding).^38^ However, in the context of classical 5-HT-induced behaviors in rodents, the (−)-enantiomer appears to be responsible for the increased neuronal uptake of 5-HT in vivo.^40,41^ The (+)-enantiomer, in contrast, was found to have marginal activity but did not appear to significantly inhibit the activity of the (−)-enantiomer. Interestingly, the “less active” (+)-enantiomer has been described as a potential treatment for memory and cognitive disorders due to its high bioavailability relative to the racemate and its lack of effect on serotonergic transmission, although further studies are certainly needed to shed light on the medicinal chemistry and pharmacology of each enantiomer. Additionally, the absolute stereochemistry of each enantiomer has yet to be defined.^31,38,42^

## Drug Metabolism and Pharmacokinetics

**Scheme 2:**
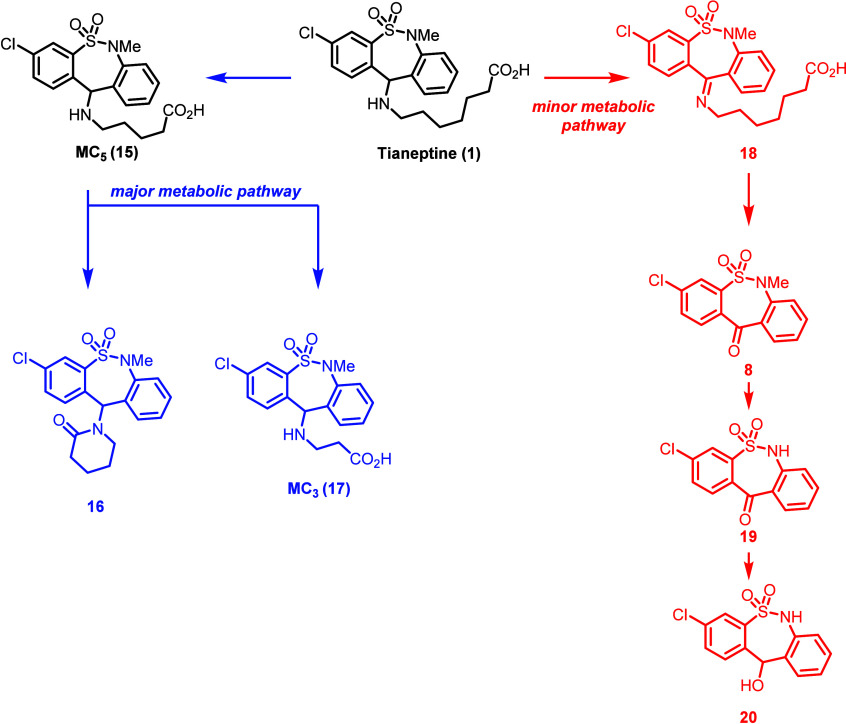
Metabolic Pathways of Tianeptine

Prescription tianeptine is administered orally. Unlike conventional TCA metabolism, tianeptine is not primarily metabolized by cytochrome P450s (CYPs), although CYP-mediated generation of reactive covalent metabolites is known.^43−50^ In one study using [^14^C]-labeled tianeptine administered to six healthy male volunteers, two primary routes of metabolism were observed (Scheme 2).^51^ The major metabolic pathway observed for tianeptine was β-oxidation of the heptanoic acid tail to a pentanoic acid tail (MC_5_, **15**). Metabolite MC_5_ underwent further β-oxidation to give a propionic tail (MC_3_, **17**). Lactonization of the MC_5_ pentanoic tail was also observed, forming the corresponding δ-lactam compound **16**. A minor metabolic pathway for tianeptine was found to be oxidation of the amine tail to give imine **18** followed by imine hydrolysis to ketone **8** (an intermediate in tianeptine synthesis), *N*-dealkylation of the methylsulfonamide to give **19**, and reduction of the ketone to give alcohol **20**. Tianeptine has been found to be extensively metabolized via the major β-oxidation pathway; 24 h after administration, the parent molecule accounted for less than 3% of the administered dose in urine with the 3 primary metabolites all products of the β-oxidation pathway. In feces and plasma samples, the metabolite profile was found to be comparable. Similar metabolic pathways have been observed in other species.^52^

After oral ingestion of a single dose of tianeptine sodium salt (12.5 mg) to 12 healthy volunteers (6 men and 6 women), the average peak plasma level (*C*_max_) was found to be 334 ± 79 ng/mL with a *T*_max_ of 0.94 ± 0.47 h. The absorption half-life was 0.19 ± 0.14 h, and the terminal half-life was 2.5 ± 1.1 h. Compared to tianeptine, the average peak plasma level for major metabolite MC_5_ was lower (63 ± 14 ng/mL) with a *T*_max_ of 2.23 ± 0.42 h and a plasma elimination half-life of 7.2 ± 5.7 h. The average systemic bioavailability of tianeptine after oral administration was high in fasting young healthy subjects (99%, with no single measured value lower than 66%).^53^ In contrast to other TCAs, tianeptine is rapidly absorbed after administration without an extensive first-pass effect.^53−57^ The same trend for relative *C*_max_, *T*_max_, and terminal half-life values for both tianeptine and MC_5_ was observed in rats after chronic (i.p.) administration.^43,58^ The influence of food on tianeptine absorption has also been studied; when tianeptine was given at the end of a meal, absorption was slightly delayed (with lower *C*_max_ and higher *T*_max_ values), although this influence was found to be marginal from a clinical standpoint.^59^ Additionally, alcohol consumption resulted in a modest reduction in absorption rate for the parent compound (30% reduction in peak plasma concentration, with a ∼20 min delay in achieving *T*_max_) but did not significantly affect plasma levels of MC_5_.^53^

## Pharmacodynamics

Early experiments suggested that tianeptine’s mechanism of action was serotonergic, although the observed enhancement of 5-HT uptake was directly opposed to the uptake inhibition associated with the SSRIs. Both acute and repeated doses of tianeptine were observed to increase 5-HT uptake without affecting the release, binding, or uptake of an array of neurotransmitters (acetylcholine, dopamine, epinephrine, GABA, glutamate, histamine, norepinephrine) along with an apparent lack of amine-oxidase activity.^60−63^ The finding that 5-HT uptake inhibitors (classical TCAs) and putative serotonin uptake enhancers (tianeptine) both show antidepressant activity continues to challenge the monoamine hypothesis of depression. Indeed, follow-up studies have suggested that an enhancement of serotonin uptake may not be the main driver of tianeptine’s antidepressant effects. Specifically, imipramine and tianeptine were found to have different actions on 5-HT_2_ and 5-HT_1A_ receptors, but both decreased [^3^H]-paroxetine binding to 5-HT transporter sites, suggesting a different mechanism for the two drugs independent of their effects on 5-HT reuptake.^64^ In addition, an in vivo microdialysis study showed that tianeptine did not change extracellular concentrations of 5-HT ([5-HT]_ext_), in contrast to the previous studies in which tianeptine inhibited the K^+^-induced increase of [5-HT]_ext_ in the ventral hippocampus and frontal cortex.^65−67^ This apparent discrepancy could imply limited resources with which to detect 5-HT in the initial studies, and a growing body of evidence suggests that an involvement of 5-HT in the efficacy of tianeptine is unlikely.^68^

Hypotheses on tianeptine’s mechanism of action have gradually shifted from serotonergic to glutamatergic. Specifically, tianeptine may be acting by influencing the expression of synaptic plasticity via the modulation of glutamatergic transmission and is known to modulate the phosphorylation state of glutamate receptors.^69,70^ In one study, long-term tianeptine administration was examined at hippocampal CA3 commissural associational (c/a) glutamate receptor ion channels (NMDA and AMPA) and was found to normalize the amplitude ratio of NMDA to AMPA/kainate receptor-mediated currents and prevent the stress-induced attenuation of NMDA-excitatory postsynaptic currents. This effect was attenuated by administration of a kinase inhibitor, suggesting that tianeptine acts via a postsynaptic phosphorylation cascade at the CA3 c/a synapse.^71^ Follow-up studies have corroborated both AMPA involvement and a postsynaptic site of action, and specific inhibitors for protein kinase A (PKA) and calmodulin-dependent protein kinase II (CaMKII) indicated that both kinases are important players in tianeptine’s effect on AMPA responses.^72^

Tianeptine has also been proposed to act as an agonist of the adenosine A1 receptor (A1R),^73^ although no direct A1R activity was observed in calcium functional assays.^74^ A screen of tianeptine across a broad panel of CNS receptors (>50 targets, Psychoactive Drug Screening Program, University of North Carolina) revealed the mu opioid receptor (MOR) as the only hit at a 10 μM screening concentration. In follow-up concentration–response experiments, tianeptine was found to be an agonist for both human and mouse MOR with weaker activity at the delta opioid receptor (DOR) and no appreciable activity for the kappa opioid receptor (KOR) (see Table 1).^74^ The compound is best characterized as a moderately potent but highly efficacious and selective MOR agonist (*E*_max_ > 100% relative to DAMGO).^74,75^ In mice, using a combination of knockout (KO) studies and pharmacological inhibition (naloxone), both the analgesic and the antidepressant effects of tianeptine were found to be MOR dependent. These results validate the involvement of MOR as an important CNS target for tianeptine and suggest that the receptor is at least partially involved in its antidepressant efficacy. Metabolite MC_5_, which is an approximately 3-fold weaker agonist at both human and mouse MOR in functional assays, mimicked the behavioral effects of tianeptine in a MOR-dependent manner. The human MOR potency for metabolite MC_3_ is very weak (EC_50_ = 16 μM) and is therefore unlikely to play a role in the observed behavioral effects. In the same study, tianeptine was not found to display the tolerance or withdrawal behaviors associated with classical opioids in mice, raising the intriguing possibility that a MOR-preferring antidepressant may be relatively safe and well tolerated.^76^ Another study suggested that tianeptine may have a lower potential to cause tolerance and dependence in rodents compared to other classical opioids of abuse while still noting MOR agonist-like acute adverse effects (constipation, respiratory depression, etc.).^77^ Case studies involving robust withdrawal symptoms after recreational use, however, cast doubt on the translatability of these findings to human subjects.

**Table 1:** Opioid Receptor Binding and Efficacy Data for Tianeptine^a^

	*K*_i_ ± SEM^b^ (nM)			EC_50_ ± SEM^c^ (nM)		
	MOR	DOR	KOR	MOR	DOR	KOR
human	383 ± 183	>10 000	NA	194 ± 70	37 400 ± 11 200	NA
mouse	NR	NR	NR	641 ± 120	14 500 ± 6600	NR

a NR = not reported, NA = no activity.

b Radioligand displacement binding.

c Functional G protein activation assay (BRET). See refs (74−76) for additional details.

## Adverse Effects and Dosage

The typical dosage of tianeptine is 12.5 mg/day three times a day for adults and 25 mg/day for the elderly.^78^ In clinical studies, a relative lack of adverse effects relative to classical TCAs has been observed.^79^ While reductions in attention and memory are common adverse effects associated with the use of TCAs, clinical studies with tianeptine have shown that these cognitive functions are largely unaffected.^80−82^ Adverse effects on sleep quality are known but largely context dependent; one study using a single dose of tianeptine (12.5 mg) reported more subjects with restless sleep versus the placebo, but another study using higher doses (37.5 mg) found sleep was more effective with tianeptine.^79,81^ In elderly populations, tianeptine had an advantage over mianserin, a tetracyclic antidepressant (TeCA), in terms of decreasing risk of falling and impaired vigilance.^83^ Additionally, another study comparing tianeptine with mianserin showed that average tianeptine doses did not have a measurable effect on driving skills.^84^

Adverse effects have also been observed through unregulated use of tianeptine and include nausea, vomiting, and abdominal pain.^85^ The case reports on tianeptine abuse vary greatly from the clinical studies and are difficult to compare due to the extremely high doses typically ingested (from 87.5 mg to 10 g).^86,18^ At least two cases of reported suicide associated with tianeptine use are known and further underscore the danger of the drug when taken at abnormally high doses.^87,88^ Further issues related to tianeptine overdose will be discussed in the Current Issues and Concerns section.

## History and Importance in Neuroscience

As a microcosm for the transition from serotonergic, to glutamatergic, to other hypotheses of depression (opioid and beyond), the tianeptine story is a fascinating case study. In the early history of antidepressants, it was thought that the TCA mechanism of action involved the uptake inhibition of either or both noradrenaline and serotonin.^89^ That tianeptine appeared to directly contradict this hypothesis while still providing relief from depression symptoms sparked renewed interest in untangling the root causes of depression and related mood disorders.

Tianeptine (Stablon) has been marketed by Laboratoires Servier in France since 1989 (approximately 20 years after the disclosure of its synthesis) and is now available in 15 countries in the European Union (France, Luxembourg, Portugal, Bulgaria, Romania, Slovakia, Poland, Malta, Hungary, Lithuania, Slovenia, Czech Republic, Austria, Latvia, and Estonia).^90^ In the decades following tianeptine’s initial launch, several countries outside the EU have approved the drug for marketing (e.g., Singapore in 1999 through license no. SIN11182P),^91^ and tianeptine is now available in at least 66 countries around the world.^90^ Tianeptine has never been marketed in the United States for reasons that predate research into its potential for abuse; the projected profits for the drug were not expected to outweigh the cost for running a clinical trial.^92^ As of 2024, however, a clinicaltrials.gov search for “tianeptine” yields 9 total studies examining the utility of tianeptine (alone or as a combination therapy) across a diverse range of indications including treatment-resistant depression, bipolar depression, postmastectomy pain after breast cancer surgery, and brain fog symptoms related to COVID-19.^93^ Additional reports have also suggested that the utility of tianeptine may extend beyond depression treatment. Tianeptine has demonstrated significant efficacy as an anxiolytic with one study noting that both tianeptine and paroxetine, an SSRI, were similarly efficacious in the 35% CO_2_ panic challenge (administration of a harmless mixture of 35% CO_2_/65% O_2_ gas which stimulates panic through chemoreceptor-mediated CO_2_ sensitivity).^94^ Tianeptine has also demonstrated efficacy as a treatment for asthma, irritable bowel syndrome (IBS), convulsions, fibromyalgia, and attention-deficit hyperactivity disorder (ADHD).^95−99^

In 2014, researchers at Columbia University demonstrated that tianeptine is an agonist for the MOR^74,76^ and accordingly produces significant antinociception in rodent pain models (corroborating similar findings conducted prior to a definitive understanding that the antinociceptive effects were MOR mediated).^100^ It is well established that MOR activation can lead to drug tolerance, dependence and abuse,^101^ and these issues were observed with tianeptine use prior to this mechanistic finding. In 2012, the Haute Autorité de Santé (French National Authority for Health) published a document aiming to reassess the benefit/risk ratio of tianeptine (Stablon) considering increasing reports of abuse and drug dependence in France. Specifically, in 2005, Stablon underwent an addiction vigilance survey which led to the addition of a warning about the risk of abuse and dependence in patient leaflets. In 2011, the National Narcotics and Psychotropics Committee found “persisting cases of tianeptine abuse and drug dependence and requested that the benefit/risk ratio of the substance be re-assessed.”^90^ As another metric to assess the abuse potential of a given substance, “doctor shopping” (the simultaneous consultation of multiple physicians) has also been associated with tianeptine in France.^102^ Ultimately, the benefit/risk ratio for tianeptine was found to be positive, although the criteria for prescribing and dispensing the drug in France have been tightened.^90^

Although tianeptine is not approved by the FDA, similar assessments are actively ongoing in the United States, where tianeptine is widely available through retailers as an unregulated supplement. Currently, outright bans on tianeptine have been enacted in at least 9 states, where the drug is officially regulated as either a schedule I or a schedule II substance.^103^ In Tennessee, bill HB2043 (2022) added tianeptine and “any salt, sulfate, free acid, or other preparation of tianeptine, and any salt, sulfate, free acid, compound, derivative, precursor, or preparation thereof that is substantially chemically equivalent or identical with tianeptine, as a schedule II controlled substance”.^104^ Reassessments of the risk associated with tianeptine extend beyond the United States and France. In Turkey, where tianeptine can be obtained without a prescription, citizens of Georgia buy the drug frequently in large quantities, and tianeptine is often referred to as “the Georgian drug” in the region (where intravenous administration is also common).^105^

## Current Issues and Concerns

Prior to 2014, few cases of tianeptine abuse were known, but reports have been increasing steadily. The FDA Center for Food Safety and Applied Nutrition (CFSAN) Adverse Event Reporting System (CAERS) received more such reports in 2022 than in the previous 3 years combined.^106^ These incidents of abuse are typically encountered either as acute effects from drug withdrawal or as death from an overdose (whether intentional or unintentional). The opioid antagonists naloxone and/or buprenorphine are often effective for overdose prevention, and several uses of this strategy have been documented.^107−109^ Recently, Tonix Pharmaceuticals patented a tianeptine oxalate/naloxone combination for the treatment of depression with a minimization of abuse potential.^110^

**Figure 6:**
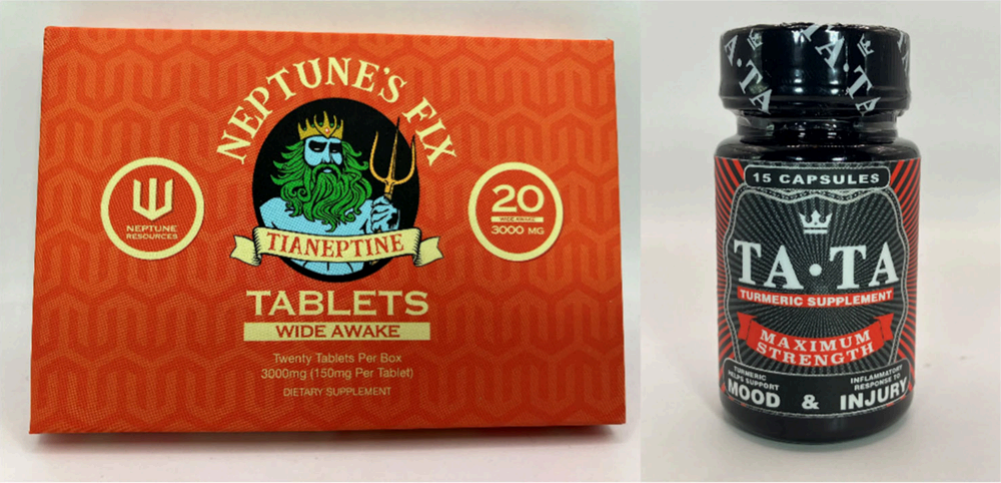
Examples of products containing tianeptine. Such products are often labeled in a misleading manner; see the “turmeric supplement” on the right-hand side. Photos courtesy of the FDA’s Office of Regulatory Affairs, Health Fraud Branch.

**Figure 7:**
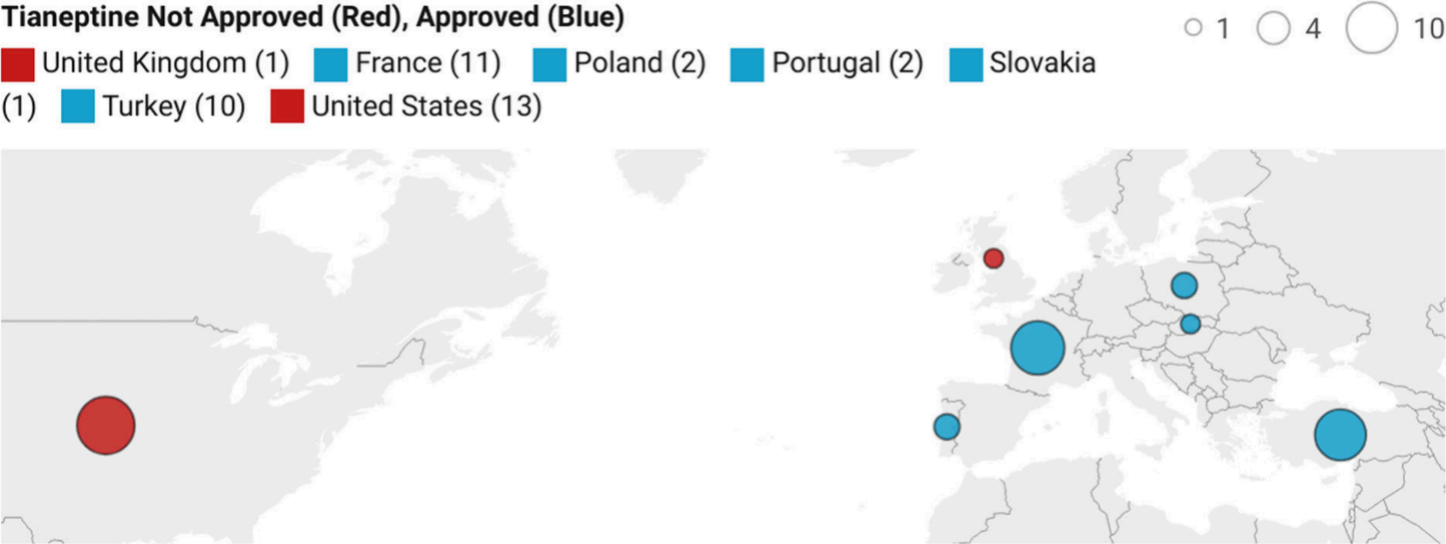
Global case reports of tianeptine abuse.^114^

In the United States, tianeptine is part of a billion dollar nootropic supplement industry and can be easily obtained in many states despite explicit warnings from the FDA against its sale and distribution (see Figure 6).^111,112^ Although tianeptine is well recognized to be an opioid by recreational users, the drug is often perceived on social media as being less dangerous than other opioids or substances of abuse and is taken in doses far beyond a typical prescription.^113^ Literature case reports paint a similar picture. In 2018, Lauhan and colleagues disclosed a thorough meta-analysis of tianeptine abuse case studies, collated through PubMed searches for “tianeptine abuse” and “tianeptine dependence”. These searches yielded 25 articles describing 65 total patients (of which more than 80% were outside the United States) taking an average dose of ∼2000 mg daily (nearly 100× times the upper dosages of typical prescriptions). Excluding the 24 patients from unreported locations in eastern Europe, the breakdown of abuse cases by specific country is summarized in Figure 7. A range of withdrawal symptoms and complications is described in these studies, and in many instances, tianeptine was taken concurrently with other substances.^114^

In one case study from the United States, a 28-year-old male was discovered unresponsive and lying on the floor of his residence. Despite a history of alcohol, tobacco and illicit drug use, he had reportedly been clean for “some time”, and indeed, a toxicology report indicated no significant evidence for substances other than tianeptine in his blood. Tianeptine itself was reported at a concentration of 2.0 mg/L, and while this number is somewhat lower than other reported fatal tianeptine concentrations, the autopsy findings of pulmonary edema and urinary retention are “suggestive of a medication toxicity with respiratory depression”. Significant amounts of metabolite MC_5_ were also identified in the TOF spectrum.^115^ These results suggest that tianeptine intoxication was the primary contributor to the cause of death.

It can be difficult to reconcile the reports of abuse and overdose with the encouraging clinical outcomes often observed for tianeptine monotherapy. In one such study, a 72-year-old female reported a long history of depression symptoms (>25 years) with >5 previous antidepressants all failing to give a positive result. This patient was given tianeptine at 12.5 mg/day for 2 years with no additional treatments, and her HDMS score improved from 28 (severe depression) to 6 (normal) over the course of the study with remission lasting out to at least 2 additional years.^116^ A subsequent report supports the idea that a MOR agonist may indeed be an effective treatment for major depressive disorder provided that the compound is administered alongside a MOR antagonist.^117^

The contrast between these case studies highlights two of the most fundamental yet often neglected tenets of medicine and toxicology: (1) that the importance of context cannot be overstated (the mindset, environment, and physiological disposition of the patient) and (2) that no compound is intrinsically toxic or nontoxic; toxicity is a function of dose.

## Conclusion

The discrepancy between tianeptine as a well-tolerated depression treatment and as a recreationally abused opioid has never been more apparent. From a basic research standpoint, tianeptine remains a useful and underutilized tool with which to (1) understand how a mechanistically differentiating TCA can elicit a positive signal in classical models of depression and (2) to specifically understand the contributions of glutamate neurotransmission and/or the MOR in these models. Structurally, the tianeptine scaffold is conducive to creative medicinal chemistry, and the pharmacology of new synthetic analogs remains a fascinating prospect (recently, structural analogs of tianeptine have been reported as class I histone deacetylase (HDAC) inhibitors).^118^ Additionally, the finding that coadministration of a MOR antagonist (samidorphan) alongside a MOR agonist (buprenorphine) can be an effective treatment for depression in patient populations raises the intriguing possibility of other coadministration or mixed-efficacy approaches.^117^ For example, MOR agonists with concomitant DOR antagonism as part of their polypharmacology have been explored as a viable strategy to attenuate the negative side effects associated with pure MOR agonists,^119−121^ although it remains to be seen how such an approach might fare in the context of depression.

Conversely, a disturbing increase in reports of recreational use, particularly in Eastern Europe and the United States, has challenged the case for the drug’s widespread availability and less restrictive scheduling. That the same molecule can be encountered as a legal antidepressant in one country and as “gas station heroin” in another speaks to the pressing need for the dissemination of accurate drug information (regardless of any stances on regulatory approval). It is our hope that this need continues to be met for tianeptine as well as for any compound with a complicated neuroscientific history.
